# Relationship Among Workplace Bullying, Job Satisfaction, Burnout, and Turnover Intention: The Mediating Role of Nurses’ Resilience

**DOI:** 10.1155/nrp/8868659

**Published:** 2026-04-24

**Authors:** Ana Cláudia Souza-Costa, Henrique Ceretta Oliveira, Edinêis Brito Guirardello

**Affiliations:** ^1^ School of Nursing, University of Campinas (UNICAMP), Campinas, São Paulo, Brazil, unicamp.br

**Keywords:** burnout, job satisfaction, nurses, psychological, resilience, workplace bullying

## Abstract

**Introduction:**

Nurses are often exposed to workplace bullying, which can result in declining job satisfaction, burnout, and a higher turnover intention. Resilience can help nurses cope with these adverse situations.

**Aim:**

To examine the relationships among workplace bullying, resilience, job satisfaction, burnout, and turnover intention in nurses and to investigate the mediating role of resilience in these correlations.

**Methods:**

This cross‐sectional study involved 239 nurses from three hospitals in the state of São Paulo, Brazil. Between April and August 2024, data were obtained via an online survey, including personal and professional information, and Brazilian versions of validated instruments for measuring resilience, workplace bullying, and burnout. Data analysis was conducted using Spearman’s correlation test and structural equation modeling.

**Results:**

Of the participants, 80.33% were female, with a mean age of 37.11 (±9.10) years. Workplace bullying was found to have a significant negative correlation with resilience, reduced personal accomplishment, and job satisfaction, as well as significant positive correlations with emotional exhaustion, depersonalization, and turnover intention. Workplace bullying had significant negative direct and indirect effects on job satisfaction, as well as significant positive direct and indirect effects on burnout. The effects of workplace bullying on job satisfaction and turnover intention were partially mediated by resilience. However, no mediating effect was found in the relationship between workplace bullying and turnover intention.

**Conclusion:**

Resilience partially mediated the relationships between workplace bullying and both job satisfaction and burnout, highlighting its protective role. However, no mediating effect was found with regard to turnover intention, suggesting that other factors influence nurses’ decisions to leave. Nurse managers can enhance professionals’ well‐being by reducing workplace bullying and implementing strategies that strengthen resilience.

## 1. Introduction

Nursing professionals constitute the largest workforce responsible for continuous patient care. Despite their crucial role in healthcare delivery, the World Health Organization estimates a global shortage of approximately 5.9 million nurses [1]. This shortage is partly due to high staff turnover [2–4] and reflects the interplay between adverse organizational conditions, particularly workplace bullying, and the availability of individual resources such as resilience [5, 6].

Workplace bullying refers to repeated and persistent negative acts, including harassment, exclusion, and abuse of power [7, 8], and represents a chronic occupational stressor in healthcare settings. Compared to other occupational groups, nurses are disproportionately exposed to such behaviors [9–11]. Within the Conservation of Resources (COR) theory [12], workplace bullying constitutes a sustained process of resource loss. Individuals are compelled to continuously invest psychological, social, and emotional resources to cope with interpersonal hostility, protect their professional identity, and maintain work performance [13]. As these resources are finite, repeated exposure to workplace bullying gradually exhausts individuals’ capacity to cope, resulting in a spiral of cumulative loss [14].

According to COR theory, stress emerges when personal resources are threatened, depleted, or insufficiently replenished [12]. In the context of workplace bullying, ongoing resource depletion can undermine nurses’ emotional energy, sense of competence, and social support, thereby increasing their vulnerability to burnout, particularly emotional exhaustion and disengagement [12, 13, 15]. As the loss spiral progresses, the erosion of valued work‐related resources, such as professional fulfillment and perceived control, further contributes to reduced job satisfaction and strengthens behavioral withdrawal responses, including turnover intention [16, 17]. Therefore, burnout and diminished job satisfaction can be conceptualized as proximal outcomes of cumulative resource loss, whereas turnover intention reflects a distal behavioral response aimed at preventing further depletion [14].

Within this theoretical framework, resilience is a key personal resource that promotes the protection and recovery of other resources. Defined as the ability to adapt and recover from stressors [18], resilience enhances individuals’ capacity to mobilize coping strategies, reinterpret adversity, and restore depleted emotional and cognitive resources [19, 20]. As such, resilience can interrupt the spirals of resource loss [14] triggered by workplace bullying, mitigating its negative effects on burnout [21, 22], job satisfaction [23], and turnover intention [24, 25]. Conversely, prolonged exposure to workplace bullying can erode resilience, reducing individuals’ ability to replenish resources and accelerating loss spirals, thereby exacerbating adverse occupational outcomes [14].

Although previous studies have demonstrated direct associations between workplace bullying and burnout [26], job satisfaction [23], and turnover intention [6], the theoretical mechanisms through which these relationships unfold are not well understood. Specifically, there is a global lack of empirical studies testing resilience as a personal resource that mediates the relationship between workplace bullying and burnout, job satisfaction, and turnover intention within the COR framework. This gap is particularly evident in the Brazilian context and among nursing professionals, where there is a lack of empirical evidence examining these associations within an integrated theoretical model. This is despite the presence of contextual and organizational characteristics that could influence exposure to workplace bullying and the availability of personal resources. Addressing these gaps is essential to advance understanding of how personal and organizational resources interact to influence occupational well‐being in nursing.

Therefore, this study aimed to examine the relationships among workplace bullying, resilience, job satisfaction, burnout, and turnover intention among nursing professionals and to investigate the mediating role of resilience within these associations.

**FIGURE 1:**
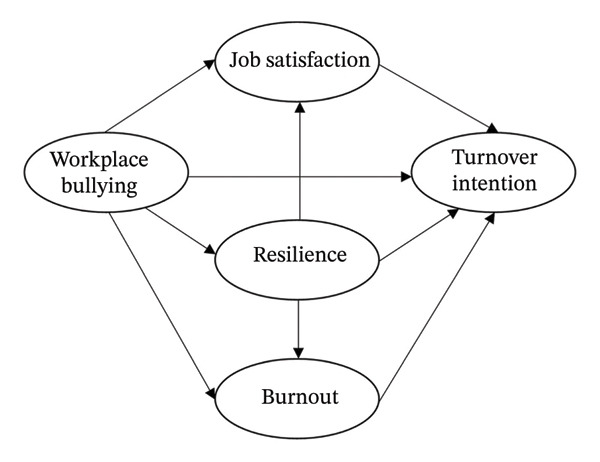
Hypothesized research model.

In this way, the proposed theoretical model of this study is illustrated in Figure 1. The hypotheses were as follows:

 Hypothesis 1: Workplace bullying is directly and positively related to burnout.
 Hypothesis 2: Workplace bullying is negatively and directly related to job satisfaction.
 Hypothesis 3: Workplace bullying is positively related to turnover intention.
 Hypothesis 4: Job satisfaction is negatively related to turnover intention.
 Hypothesis 5: Burnout is positively related to turnover intention.
 Hypothesis 6: Resilience has a mediating effect between workplace bullying and job satisfaction.
 Hypothesis 7: Resilience has a mediating effect between workplace bullying and burnout.
 Hypothesis 8: Resilience has a mediating effect between workplace bullying and turnover intention.

## 2. Methods

### 2.1. Study Design and Setting

This cross‐sectional study was carried out in three private tertiary hospitals in the state of São Paulo, Brazil.

### 2.2. Sample

The convenience sample consisted of 239 nursing professionals, including those providing direct patient care and those in supervisory roles. Professionals with 6 months or less of experience at the hospital, and those on vacation or other types of leave during the data collection period, were excluded. In the context of structural equation modeling (SEM), a sample size greater than 200 was considered adequate [27].

### 2.3. Measures

#### 2.3.1. Personal and Professional Data

The participants’ characteristics included age, gender, marital status, professional category, length of professional experience, length of experience at the hospital, work shift, and number of employees. A single‐item measure was used to assess turnover intention: *‘What is your intention to leave the job in the next year?*’ This was scored on a 10‐point Likert scale, with lower scores indicating lower turnover intention.

#### 2.3.2. Connor‐Davidson Resilience Scale (CD‐RISC‐10)

The CD‐RISC‐10 validated for Brazil [28] was used to assess resilience. The scale is unifactorial and comprises ten items. Each item is rated on a 5‐point Likert scale, ranging from 0 (*not true*) to 4 (*true almost always*). The total score can range from 0 to 40, with higher scores indicating a higher level of resilience [29]. Cronbach’s alpha was reported as 0.82 in a previous study [28]. In our study, the scale had a Cronbach’s alpha of 0.84.

#### 2.3.3. Negative Acts Questionnaire—Revised (NAQ‐R)

Workplace bullying was assessed using the Brazilian version of the NAQ‐R [30], which describes certain negative behaviors in the workplace. The 22‐item questionnaire has four subscales: Exclusion (8 items), Harassment (7 items), Quality/Overload (4 items), and Undervaluation (3 items) [31]. This tool consists of a 5‐point Likert scale, with responses ranging from 1 (*never*) to 5 (*daily*), including the categories: never, occasionally, monthly, weekly, and daily. Higher scores indicate a higher level of exposure to bullying. Participants who reported experiencing negative acts on a daily or weekly basis during the previous 6 months were considered to have suffered workplace bullying. The reliability and validity of this questionnaire were confirmed in a previous study [32]. In this study, Cronbach’s α for the four subscales ranged from 0.71 to 0.88.

#### 2.3.4. Job Satisfaction Subscale of the Safety Attitudes Questionnaire (SAQ)—Short Form 2006

Job satisfaction was assessed using the Brazilian version of the SAQ Job Satisfaction subscale [33], which consists of five items on a 5‐point Likert scale, ranging from *disagree strongly* to *agree strongly*. The scoring procedure is defined as follows: *disagree strongly* corresponds to 0 points, *disagree slightly* to 25 points, *neutral* to 50 points, *agree slightly* to 75 points, and *agree strongly* to 100 points. The total score is obtained by summing the responses to all the items in the domain and dividing the result by the number of items. The score for each subscale varies from 0 to 100. A score of 75 or higher is considered positive for job satisfaction [33]. The Brazilian version of the scale demonstrates strong evidence of validity, reliability, and responsiveness [34]. In our study, Cronbach’s alpha for the SAQ was 0.84.

#### 2.3.5. Maslach Burnout Inventory (MBI)

The Brazilian version of the MBI was used to measure levels of burnout [35]. Consisting of 22 items, it has three dimensions: Emotional Exhaustion (9 items), Depersonalization (5 items), and Reduced Personal Accomplishment (8 items). Responses are given on a five‐point Likert scale, ranging from 1 (*never*) to 5 (*every day*). The emotional exhaustion dimension ranges from nine to 45 points, while the depersonalization dimension ranges from five to 25 points. For both dimensions, higher scores indicate higher levels of burnout. In contrast, the reduced personal accomplishment dimension ranges from eight to 40 points, with lower scores, reflecting higher levels of burnout [36]. Burnout syndrome is defined by high scores in emotional exhaustion and depersonalization and low scores in reduced personal accomplishment. Previous research has confirmed the reliability and validity of the MBI [37]. In this study, Cronbach’s alpha was 0.93 for emotional exhaustion, 0.70 for depersonalization, and 0.80 for reduced personal accomplishment.

### 2.4. Data Collection

Online data collection was conducted between April 24, 2024, and August 9, 2024. The survey instruments were entered into the Google Forms platform, and a link and QR code were made available to access them. Invitations to take part in the survey were sent via institutional email, posters, and social media. All participants gave their consent before answering the questions and downloading a copy of the informed consent form upon clicking on the link.

### 2.5. Data Analysis

Data were examined using the Statistical Analysis System (SAS) program, Version 9.4, and SmartPLS 4 [38]. Descriptive statistics, with mean, standard deviation, and frequency, were used to analyze personal and professional characteristics. Spearman’s correlation coefficient was used to analyze the correlation between the variables.

We analyzed the proposed theoretical model using a SEM with partial least squares (PLS) as the estimation method [39]. Model estimates were obtained using the bootstrap method with 5000 samples and 95.0% confidence intervals (CIs) to test the direct and indirect effects. There is complete mediation when the indirect effect is statistically significant and the direct effect is not. Partial mediation occurs when the direct and indirect effects are significant, and there is no mediating effect when the indirect effect is not significant [39]. When partial mediation is observed, the direct and indirect effects are multiplied. A result with a positive sign for this multiplication indicates that there is complementary partial mediation, and a result with a negative sign indicates that there is competitive partial mediation [39].

Pearson’s coefficient of determination (*R* ^2^) was used to evaluate the quality of the structural model. The model was classified as having a small effect (2.0%), a medium effect (13.0%), or a large effect (26.0%). Additionally, the predictive validity coefficient (*Q* ^2^) was considered adequate if it was greater than zero. The effect size (*f* ^2^) was also used to evaluate the change in *R* ^2^ when an exogenous construct was omitted from the model. Values equal to 0.02, 0.15, and 0.35 represent small, medium, and large effects, respectively [39].

### 2.6. Ethical Considerations

The study received approval from the university’s Ethics Committee (Number: 6.718.015) before the start of the research. Informed Consent was obtained from all participants. All information gathered was handled in a strictly confidential manner.

## 3. Results

### 3.1. Demographic, Occupational, and Organizational Characteristics of the Participants

A total of 239 nursing professionals participated in this study, with a mean age of 37.11 (±9.10) years. The average length of nursing experience was 12.23 (±7.05) years, and in the institution, it was 6.22 (±5.73) years. The majority of nurses (74.48%) had no other employment. Other characteristics of the participants are presented in Table 1.

**TABLE 1:** Demographic and occupational characteristics of participants (*n* = 239).

Variable	*n*	%
Gender		
Female	192	80.33
Male	45	18.83
Chose not to provide information	2	0.84
Marital status		
Unmarried	124	51.88
Married	114	47.90
Did not provide information = 1		0.22
Work shift		
Day shift	154	64.43
Night shift	79	33.05
Did not provide information = 6		2.52

### 3.2. Characteristics of Workplace Bullying, Resilience, Burnout, Job Satisfaction, and Turnover Intention

The nurses presented a mean score of 33.21 (±12.51) for workplace bullying. Of the participants, 20 (8.37%) reported never having experienced workplace bullying, 197 (82.43%) reported experiencing it occasionally, and 22 (9.21%) frequently. Additionally, the mean scores were 27.12 (±5.84) for resilience, 24.70 (±7.96) for emotional exhaustion, 30.52 (±4.63) for reduced personal accomplishment, and 9.44 (±3.77) for depersonalization. Regarding job satisfaction, participants’ average score was 72.49 (±22.28). Additionally, the professionals showed no intention of leaving their jobs within the next year (3.80 ± 3.86).

### 3.3. Correlations Among Variables

Table 2 shows the results of analyzing the correlation among workplace bullying, resilience, job satisfaction, burnout, and turnover intention.

**TABLE 2:** Spearman’s correlations among resilience, workplace bullying, burnout, job satisfaction, and turnover intention.

Variable	1	2	3	4	5	6	7	8	9	10
1. Resilience	1									
2. WB: exclusion	−0.335^∗^	1								
3. WB: harassment	−0.245^∗∗^	0.703^∗^	1							
4. WB: quality/overload	−0.236^∗∗^	0.656^∗^	0.612^∗^	1						
5. WB: undervaluation	−0.230^∗∗^	0.608^∗^	0.577^∗^	0.634^∗^	1					
6. Burnout: emotional exhaustion	−0.428^∗^	0.485^∗^	0.415^∗^	0.561^∗^	0.458^∗^	1				
7. Burnout: reduced personal accomplishment	0.520^∗^	−0.251^∗^	−0.195^∗∗^	−0.289^∗^	−0.308^∗^	−0.596^∗^	1			
8. Burnout: depersonalization	−0.291^∗^	0.456^∗^	0.399^∗^	0.515^∗^	0.462^∗^	0.582^∗^	−0.421^∗^	1		
9. Job satisfaction	0.430^∗^	−0.481^∗^	−0.379^∗^	−0.473^∗^	−0.434^∗^	−0.684^∗^	0.518^∗^	−0.489^∗^	1	
10. Turnover intention	−0.155^∗∗^	0.285^∗^	0.193^∗∗^	0.308^∗^	0.362^∗^	0.471^∗^	−0.243^∗^	0.330^∗^	−0.475^∗^	1

Abbreviation: WB, workplace bullying.

^∗^ *p* < 0.0001.

^∗∗^ *p* < 0.01.

### 3.4. SEM

The estimated effects for the hypothesized structural paths are shown in Table 3.

**TABLE 3:** Estimates of the effects for the proposed structural paths.

Structural paths	Standardized coefficients (β)	95% CI		*p*
		LL	UL	
Workplace bullying ⟶ resilience	−0.295	−0.437	−0.177	< 0.0001
Resilience ⟶ job satisfaction	0.350	0.243	0.466	< 0.0001
Resilience ⟶ burnout	−0.391	−0.489	−0.297	< 0.0001
Resilience ⟶ turnover intention	0.111	−0.028	0.247	0.1127
Workplace bullying ⟶ job satisfaction	−0.386	−0.483	−0.283	< 0.0001
Workplace bullying ⟶ burnout	0.419	0.325	0.497	< 0.0001
Workplace bullying ⟶ turnover intention	−0.020	−0.171	0.124	0.7969
Job satisfaction ⟶ turnover intention	−0.409	−0.566	−0.253	< 0.0001
Burnout ⟶ turnover intention	0.173	−0.005	0.352	0.0599

Abbreviations: LL, lower limit; UL, upper limit.

**FIGURE 2:**
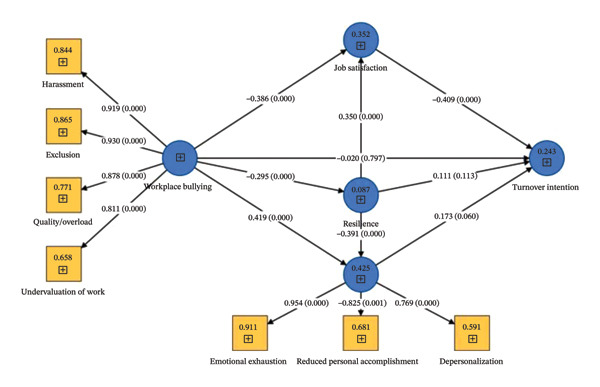
Structural equation modeling results.

The SEM model (Figure 2) indicates a significant negative effect of workplace bullying on job satisfaction (*β* = −0.386, 95% CI [−0.483, −0.283]) and a positive effect on burnout (*β* = 0.419, 95% CI [0.325, 0.497]). Thus, Hypotheses 1 and 2 were confirmed.

In addition, job satisfaction had a significant negative effect on turnover intention (*β* = −0.409, 95% CI [−0.566, −0.253]), confirming Hypothesis 4. On the other hand, Hypotheses 3 and 5 were not confirmed, suggesting that neither workplace bullying nor burnout had a significant effect on turnover intention (*β* = −0.020, 95% CI [−0.171, 0.124]) (*β* = 0.173, 95% CI [−0.005, 0.352]).

As shown in Table 4, *R* ^2^, *Q* ^2^, and *f* ^2^ were used to evaluate the structural model. The *R* ^2^ values were large for job satisfaction and burnout, medium for intention to leave, and small for resilience. The *Q* ^2^ values indicated an adequate model fit, while the *f* ^2^ values indicated medium and small effects.

**TABLE 4:** Evaluation of structural model.

Variable	*R* ^2^	*Q* ^2^	*f* ^2^
Resilience	0.09	0.07	
Job satisfaction	0.35	0.23	
Burnout	0.43	0.28	
Turnover intention	0.24	0.05	
Workplace bullying ⟶ resilience			0.10
Workplace bullying ⟶ job satisfaction			0.21
Workplace bullying ⟶ burnout			0.28
Workplace bullying ⟶ turnover intention			0.01
Resilience ⟶ job satisfaction			0.17
Resilience ⟶ burnout			0.24
Resilience ⟶ turnover intention			0.01
Job satisfaction ⟶ turnover intention			0.09
Burnout ⟶ turnover intention			0.02

Resilience was also tested as a mediator in the association between workplace bullying and job satisfaction, burnout, and turnover intention (Table 5). Workplace bullying had significant negative direct and indirect effects on nurses’ job satisfaction, as well as significant positive direct and indirect effects on burnout. This indicates that resilience partially mediated the relationship between bullying and job satisfaction and between bullying and burnout, supporting Hypotheses 6 and 7.

**TABLE 5:** Direct and indirect effects for the SEM model (*n* = 239).

Variable	Direct effects	CI 95%		*p*	Indirect effects	CI 95%		*p*	Total effect	Direct effects ∗ indirect effects	Type of mediation
		LL	UL			LL	UL				
WB ⟶ JS (resilience)	−0.386	−0.483	−0.283	< 0.0001	−0.103	−0.164	−0.060	0.0001	−0.489	0.039	Complementary partial mediation
WB ⟶ B (resilience)	0.419	0.325	0.497	< 0.0001	0.116	0.066	0.181	0.0001	0.533	0.048	Complementary partial mediation
WB ⟶ TI (resilience)	−0.020	−0.171	0.124	0.7969	−0.033	−0.086	0.008	0.1667	−0.053	0.00	No mediation

Abbreviations: B, burnout; CI, confidence interval; JS, job satisfaction; LL, lower limit; TI, turnover intention; UL, upper limit; WB, workplace bullying.

The ratio of the mediating effect to the total effect for workplace bullying, job satisfaction, and resilience was 21.06%. For workplace bullying, burnout, and resilience, this ratio was 21.68%. For bullying, turnover intention and resilience, the ratio was 62.26%. However, resilience did not mediate the relationship between workplace bullying and turnover intention because the direct and indirect effects were not statistically significant; therefore, Hypothesis 8 was not supported.

## 4. Discussion

This study examined the relationships among workplace bullying, resilience, job satisfaction, burnout, and turnover intention among nurses, as well as the mediating role of resilience within these associations. The findings revealed that most participants reported exposure to occasional workplace bullying, accompanied by moderate resilience and emotional exhaustion, high depersonalization and reduced personal accomplishment, and job dissatisfaction, while turnover intention remained relatively low.

Consistent with the COR theory, workplace bullying was directly and positively associated with burnout and negatively associated with job satisfaction. As a chronic interpersonal stressor, workplace bullying compels nurses to continuously invest emotional, cognitive, and social resources to protect their professional identity, manage interpersonal conflict, and sustain work performance [14, 40, 41]. Given the finite nature of these resources, persistent exposure accelerates cumulative loss spirals [14], resulting in emotional exhaustion, job dissatisfaction, and reduced professional fulfillment [13, 42–44]. These findings reinforce the idea that workplace bullying is not merely an episodic stressor but a sustained process that depletes essential work‐related resources for well‐being [45, 46]. This highlights the importance of organizational strategies that reduce exposure to stressors and actively promote resource acquisition through supportive leadership, adequate staffing levels, and recognition practices that restore professional value and social support [47, 48].

In this sample, workplace bullying and burnout were not directly associated with turnover intention. This suggests that nurses’ turnover intention may be more strongly influenced by contextual conditions and professional commitment than by isolated occupational stressors [49]. Furthermore, a meta‐analysis indicates that the effect of burnout on turnover intention tends to be weaker among nurses than in other occupational groups [50].

Additionally, the data showed that job satisfaction was negatively associated with turnover intention, reinforcing its protective role as a key motivational and affective resource. Organizational strategies focused on workload regulation, recognition, transparent communication, and access to adequate material and human resources may therefore indirectly reduce turnover intention by sustaining job satisfaction [51–53].

A central contribution of this study lies in demonstrating that resilience partially mediates the relationships between workplace bullying, job satisfaction, and burnout. Within COR theory, resilience is a personal resource that can help interrupt loss spirals and support the replenishment of resources [14]. Nurses with higher levels of resilience are better able to mobilize adaptive strategies to cope with adversity and to restore depleted emotional and cognitive resources [54]. This can attenuate the negative effects of workplace bullying on psychological strain and professional fulfillment [55].

In COR terms, insufficient resilience can hinder an individual’s ability to replenish their resources, intensifying the cycle of cumulative losses [12–14] and increasing susceptibility to job dissatisfaction [56] and burnout [57]. Indeed, empirical data demonstrate that resilience is a critical protective factor against nurses’ burnout [58], acting as a buffer that mitigates the impact of occupational stressors. Furthermore, it significantly facilitates recovery from adverse work experiences [59, 60], enabling individuals to regain psychological equilibrium after periods of professional strain.

These findings extend prior research by empirically supporting resilience as an active mechanism that interrupts cumulative loss spirals, rather than merely correlating with positive outcomes [14]. Nevertheless, the partial nature of the mediation indicates that individual coping capacity alone is insufficient to neutralize the harmful effects of workplace bullying, emphasizing the need for systemic interventions [61].

In this context, the results also help explain why turnover intention did not emerge as a significant direct outcome of workplace bullying. It is likely that mediating and moderating mechanisms, such as burnout, organizational commitment, and resilience, play a more central role in shaping nurses’ turnover intention. From a practical perspective, these findings underscore the importance of integrated organizational strategies that simultaneously reduce resource loss and promote resource gain. Evidence‐based actions include the implementing of formal antibullying policies, confidential and nonpunitive reporting systems, and leadership training focused on relational justice and supportive supervision [62]. In parallel, resilience‐building programs, peer support groups, access to mental health services, regular workload assessments, and recognition practices can foster psychologically safe environments, enhance coping resources, and contribute to the long‐term retention of nursing staff.

### 4.1. Strengths and Limitations

This study has several limitations, one of which is the use of a convenience sample. The cross‐sectional design also limits the ability to draw inferences about causal relationships between the study variables. Furthermore, online data collection makes it difficult to control environmental factors. The sample was composed exclusively of nursing professionals from three private hospitals in a single Brazilian state, so the findings may not be applicable to other healthcare settings, such as public institutions or hospitals in different regions. Future studies could address these limitations by adopting longitudinal designs, which would facilitate the establishment of causal relationships, allow for better control of potential biases, and improve the generalizability of the findings. Additionally, future research should examine the impact of intervention programs aimed at reducing workplace bullying and strengthening resilience in clinical settings. Investigating this model in public healthcare institutions and diverse cultural or regional contexts would also provide valuable insights into its broader applicability. Furthermore, future studies could examine how potential moderating variables influence the relationships among workplace bullying, burnout, job satisfaction, resilience, and turnover intention.

## 5. Conclusions

This study demonstrates that resilience has a significant role in mediating the relationship between workplace bullying and both job satisfaction and burnout, highlighting its protective psychological resource in the nursing work environment. However, no mediating effect was observed in the relationship between workplace bullying and turnover intention, suggesting that other factors may influence nurses’ decisions to leave their jobs. These findings emphasize the importance of organizational strategies focused on preventing workplace bullying and promoting resilience to support the well‐being and retention of nursing professionals.

## Author Contributions

Study design, data collection, data analysis, and writing: Ana Cláudia Souza‐Costa.

Study design, data collection, data analysis, and writing: Edinêis Brito Guirardello.

Data analysis and writing: Henrique Ceretta Oliveira.

Review and editing: All the authors.

## Funding

This research was supported by the National Council for Scientific and Technological Development, 310130/2022‐0; Post‐Doctoral Researcher Program (PPPD) of the State University of Campinas.

## Disclosure

All authors gave the final approval of the manuscript.

## Conflicts of Interest

The authors declare no conflicts of interest.

## Data Availability

The data that support the findings of this study are available upon request.
